# Bone Regeneration Using Bio-Nanocomposite Tissue Reinforced with Bioactive Nanoparticles for Femoral Defect Applications in Medicine

**Published:** 2020

**Authors:** Mohammad Ali Maghsoudlou, Ehsan Nassireslami, Saeed Saber-Samandari, Amirsalar Khandan

**Affiliations:** 1. Department of Pharmacology and Toxicology, AJA University of Medical Sciences, Tehran, Iran; 2. New Technologies Research Center, Amirkabir University of Technology, Tehran, Iran

**Keywords:** Bone regeneration, Chitosan, Tissue engineering, Zirconium

## Abstract

**Background:**

In recent years, the method of constructing and evaluating the properties of polymer nanocomposite and bioactive ceramics in tissue engineering such as biocompatible scaffolds was studied by some researchers.

**Methods:**

In this study, the bio-nanocomposite scaffolds of Chitosan (CS)–Hydroxyapatite (HA)–Wllastonite (WS), incorporated with 0, 10, 20 and 30 wt% of zirconium were produced using a freeze-drying method. Also, the phase structure and morphology of scaffolds were investigated using X-ray Diffraction (XRD), Scanning Electron Microscopy (SEM) and Energy Dispersive Spectroscopy (EDS). By analyzing the SEM images, the porosity of the scaffolds was observed in the normal bone area of the body. In the next step, bioactivity and biodegradability tests of the scaffolds were carried out. Due to the presence of hydrophilic components and the high-water absorption capacity of these materials, the bio-nanocomposite scaffolds were able to absorb water properly. After that, the mechanical properties of the scaffolds were studied.

**Results:**

The mechanical test results showed that the preparation of reinforced bionanocomposites containing 10 wt% of zirconium presented better properties compared to incorporated bio-nanocomposites with different loadings of zirconium.

**Conclusion:**

According to MTT assay results, the prepared scaffolds did not have cytotoxicity at different concentrations of scaffold extracts. Consequently, the investigated scaffold can be beneficial in bone tissue engineering applications because of its similarity to natural bone structure and its proper porosity.

## Introduction

In the recent decade, due to various clinical bone diseases such as bone infections, bone tumors, and bone loss, the need for bone regeneration is growing 1. Common treatments for these defects are the use of autograft, allograft, xenograft and other artificial substitutes such as metals and synthetic cement 2. These alternatives are not ideal treatments and they have their limitations. For example, an autograft is associated with problems such as donor deficiency. Allograft and xenograft have the risk of transmitting disease and immune response. A major component of tissue engineering in bone regeneration is a scaffold that serves as a template for cellular interactions and the formation of an extracellular matrix for structural support of newly formed tissue. Scaffolds for bone regeneration should contain certain criteria for mechanical properties similar to those of the bone, biocompatibility, and biodegradability at a rate appropriate for regeneration 3–5.

The study of biomaterials and the method of producing scaffolds to optimize the integrity of produced bone with surrounding tissues and bones was done extensively 6–10. For instance, Tripathi *et al* 11 fabricated and characterized bio-composite scaffolds containing CS-HA and Cu-Zn alloy nanoparticles using freeze-drying technique. They observed that the addition of Cu-Zn in the CS/HA scaffolds significantly increased swelling, decreased degradation, increased protein adsorption, and increased antibacterial activity 11–15. Also, fabricated scaffolds had no toxicity towards rat osteoprogenitor cells. Yu *et al* 16 fabricated a composite scaffold made of CS, HA, alginate, and collagen using electrospinning techniques. They characterized the distribution of each component, the morphology and microstructure of the scaffold using a confocal laser scanning microscope, a field-emission scanning electron microscope and transmission electron microscopy. Their results showed that their composite was expected to be a potential scaffold for bone tissue engineering applications. Jin *et al* 17 prepared porous Chitosan-Hydroxyapatite (CS/HA-alginate composite scaffolds through *in situ* co-precipitation and freeze-drying for bone tissue engineering. In their case study, they observed that by increasing the content of HA, the porosity of the scaffolds decreased from 84.98 to 74.54%. Also, an MTT assay indicated that the obtained scaffolds did not have cytotoxic effects on MG-63 cells. Also, the obtained scaffolds showed a good biocompatibility. Sahmani *et al* 18 fabricated bioactive nanoclay-TiO_2_ bionanocomposite scaffolds containing different weight fractions of TiO_2_ *via* the space holder technique. They reported that mechanical properties of fabricated bionanocomposites including compressive strength, elastic modulus and crystallite size were 5.74 MPa, 438 MPa and 70–120 *nm*, respectively. Khandan *et al* studied the mechanical and biological properties of the bredigitemagnetite (Ca_7_MgSi_4_O_16_-Fe_3_O_4_) nanocomposite with various amounts of magnetite (0, 10, 20 and 30 wt%). According to their results, the properties of the constructed scaffolds had an extreme dependence on the magnetite content. In their case study, the optimum sample with bredigite-30 wt% magnetite showed that the fracture toughness was 2.69 MPa m^1/2^ and the elastic modulus was 29 GPa. Also, increasing bredigite content led to an increase in pH values in the SBF solution.

As the above literature overview indicates, there is no specific investigation on CS, HA, and Zirconium (Zr) bio-nanocomposite scaffolds with various amounts of zirconium (0, 10, 20 and 30 wt%) fabricated *via* novel freeze-drying technique. Zirconium can be a suitable candidate for medical use due to its ideal mechanical properties such as high strength and corrosion resistance, low cytotoxicity and magnetic sensitivity 19. To characterize the morphology and structure of mentioned bio-nanocomposite, scanning electron microscopy and X-ray diffraction analysis were used. The mechanical properties of the scaffolds were examined using tensile tests. Furthermore, Simulated Body Fluid (SBF) and physiological saline solutions were used to evaluate the feasibility of the sample for bioactive bone tissue engineering application. Finally, cytotoxicity of the scaffolds was tested by an MTT assay.

## Materials and Methods

### Experimental procedures

***Materials:*** In this study, polymer powder of CS was purchased from Aldrich Co., USA and acetic acid (CH_3_COOH, purity >99%) for the preparation of polymer solution was purchased from Ameretat Shimi Co., Iran. HA was supplied by CAM Bioceramics Co., Netherlands, and zirconium nanopowder (purity >95%) was prepared by ASEPE Co., Iran. Wollastonite (WS) (purity >99%) was purchased from ESPADANA Co., Iran. Double distilled water was also used to dissolve the polymer.

### Synthesis of bio-nanocomposite scaffolds

Nanocomposite scaffolds were prepared in two stages. They included (I) preparation of nanocomposite powder, and (II) preparation of polymer solution and dissolution of nanocomposite powder following freeze-drying. Initially, the certain weight percent of zirconium was selected as zero, 10, 20 and 30 wt%. For each sample, 2.5 *g* of hydroxyapatite and 0.2 *g* of WS were weighted using a digital scale with a precision of one-thousandth of a *g* and then the desired amount of zirconium was added.

The CS polymer powder was dissolved in twice-distilled water and then acetic acid was added to the solution to improve the polymerization and then stirred for 3 *hr* at 60*°C* using magnetic stirrer. After dissolving nanocomposite powder in the polymer solution to a uniform distribution of powder, the mixture of hydroxyapatite and zirconium was stirred for 10 *min* in a mechanical stirrer and then sonicated with 100% amplitude using an ultrasonic bath for 5 *min*. Afterward, to solidify the solid-liquid phase, the molds were placed in a freezer (FD-10, DORSA-TECH Engineering Co., Iran) at −65*°C* for 48 *hr*. Finally, samples were produced after 48 *hr* of keeping in the freeze dryer.

### Morphology and microstructure evaluation

In this study, the scanning electron microscope (SERON AIS2100) with the EDS equipment was used to study the morphology and porosity of fabricated nanocomposites. To prepare specimens for Scanning Electron Microscopy (SEM) analysis, a thin layer of gold was placed on the surface of the specimen. The X-ray diffraction analysis with 40 *kV* accelerator voltages and 30 *mAh* with a resolution of 0.1*°* (INEL EQUNIOX 3000) was also performed.

### Mechanical properties (Tensile strength and porosity)

The mechanical properties of the samples were investigated using tensile tests (SANTAM STM-50). The tensile strength was evaluated at a strain rate of 0.5 *mm/min*. The elastic modulus of the scaffold was determined from the slope of the elastic area of the diagram. To evaluate the porosity range, ImageJ software was used. To measure at least twenty cavities of each sample, the SEM images were examined.

### Biological properties

In this work, the SBF 20 was used to estimate the bioavailability of porous scaffolds. Samples were placed in a bowl containing SBF solution. The containers were incubated at 37*°C* for 14 days. The pH of container was investigated after the first, third, seventh and fourteenth days.

Furthermore, to evaluate the biodegradability of the samples, a solution of Phosphate Buffered Saline (PBS) (Sigma Aldrich, US) was used which had a pH of 7.4 at ambient temperature. Samples were stored in a container containing PBS solution for 14 days. On the first, third, seventh and fourteenth days, the samples were removed from the solution and their weight loss was measured after complete drying using the freeze-drying technique. The weight loss of the samples was obtained using below equation 21:

$$\text{weight}\;\text{loss}\;\left(\%\right)=\frac{\left({\text{w}}_{0}-{\text{w}}_{\text{d}}\right)}{{\text{w}}_{0}}\times 100$$

where W_0_ and W_d_ are the weights of the swollen and the dried scaffolds, respectively.

To evaluate the cytotoxicity of bio-nanocomposite scaffolds, the 3- (4,5-dimethylthiazol-2-yl)-2,5-diphenyltetrazolium bromide (MTT, Aldrich) assay based on extraction method was used 22. Initially, Human fibro-blast cells (HuGu) cells which were taken from gums of a 45-year old female in the Iranian National Center for Biological Resources were cultured in a fresh growth medium containing 89% high-glucose Dulbecco’s Modified Eagle’s Medium (DMEM), 2 *mmol.L*^−1^ glutamine (Biochrom, UK), 15% FBS and 1% penicillin (Merck, Germany). Cells were placed in an incubator with a 5% CO_2_ atmosphere at 37*°C* and fed every 3 days. Subsequently, 5000 HuGu cells and 100 *μl* of culture medium were poured into a 96-well culture plate and then incubated at 37*°C* for 24 *hr* to keep the cells sticking to the plate. After seeding HuGu cells, they were treated with seven concentrations of scaffolds ranging from 0.03 to 2.00 *mg.ml*^−1^ and incubated for 24, 48 and 72 *hr*. After the aspiration of the culture medium, 20 *μl* of MTT solution was poured into each well and placed in an incubator for 4 *hr*. Then, the upper solvent of wells was removed and formazan precipitate was solubilized with 200 *μl* of the DSMO for 6 *hr*. Finally, by using an absorbance microscope reader, optical density properties were recorded at 570 *nm*. For more precision, this process was repeated twice for each concentration of the scaffold. The cell viability was calculated as follows:

$$\text{Cell}\;\text{viability}\;(\%)=\frac{{\text{OD}}_{\text{sample}}}{{\text{OD}}_{\text{control}}}\times 100$$

## Results

To characterize the morphology and structure of mentioned bio-nanocomposite, SEM and X-ray Diffraction (XRD) were used. The mechanical properties of the scaffolds were examined using tensile tests. Furthermore, SBF and water solutions were used to evaluate the feasibility of the sample for bioactive bone tissue engineering applications. Finally, cytotoxicity of the scaffolds was tested by an MTT assay.

### Scanning electron microscopy analysis

**Figure 1.**
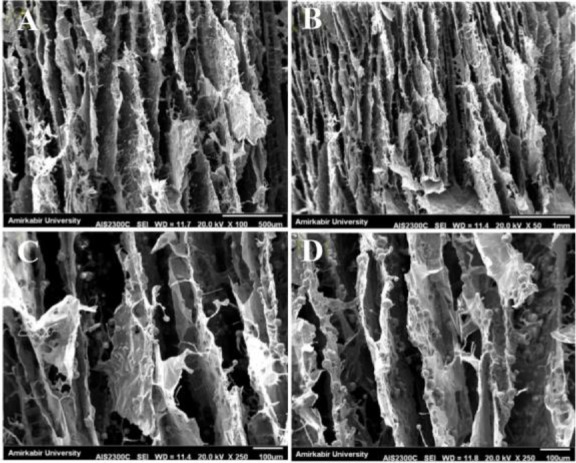
SEM images of (A) 0, (B) 10, (C) 20, and (D) 30 wt% of Zr in the CS-HA before soaking in the simulated body fluid.

The results of SEM images of fabricated scaffolds representing surface morphology at magnifications of 100 and 1000 times are shown in figure 1(A–D) before soaking in the SBF solution. In the SEM analysis, the morphology of the fabricated porous scaffolds was studied. Figure 1(A–D) shows the SEM images of specimens with a hexagonal shape. In the microscopic images of scaffolds containing nanoparticles, it was observed that adding zirconium nanoparticles to the scaffold compared to the scaffold without a nanoparticle provides a surface with less distortion. The smoothness of the surface of the cavity walls plays an important role in the adhesion and growth of the cell within the scaffold. Adding nanoparticles to the scaffold changes the structure of the scaffold. Also, it might affect the porosity and mechanical features of the architecture.

**Figure 2.**
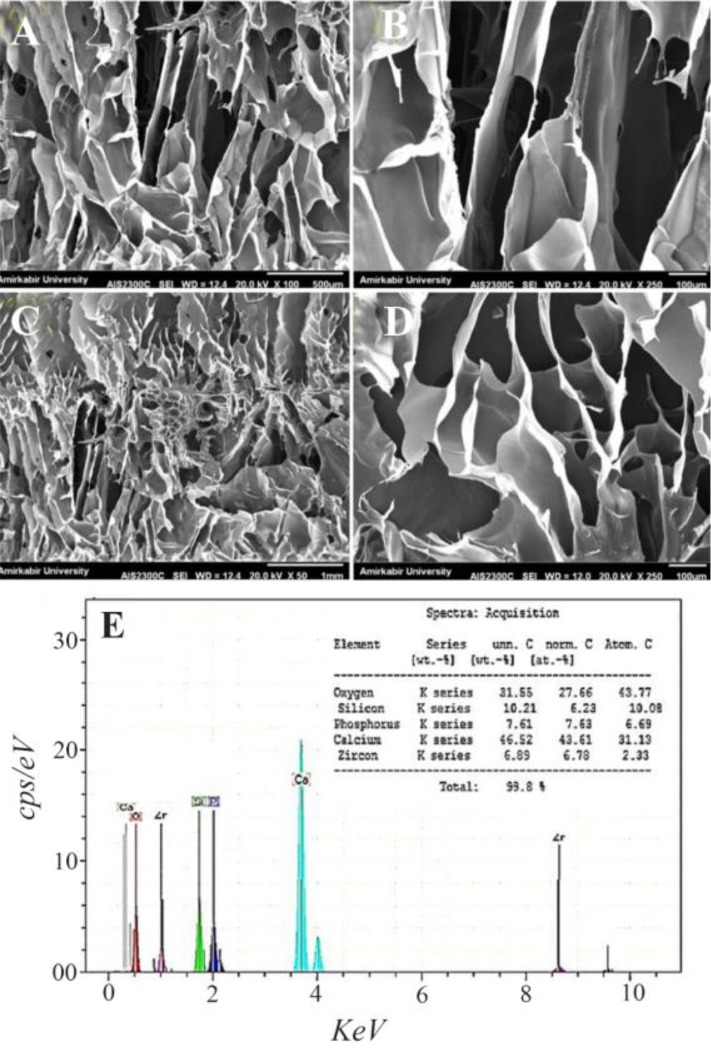
SEM images of (A) 0, (B) 10, (C) 20, and (D) 30 wt.% of zirconium in the CS-HA after soaking in the simulated body fluid and (E) corresponding EDS analysis for sample with 10 wt% of Zr.

Figure 2(A–D) shows the SEM images of the samples which were soaked in the SBF solution for 14 days in the water bath at 37*°C*. Also, the Energy Dispersive Spectroscopy (EDS) spectrum in figure 2E proves that the manufactured scaffold is composed of CS, HA, and WS containing Zr nanoparticles. The four scaffolds produced by the freeze-drying technique in this study show a surface porosity of over 70%. The results are listed in table 1. In bioengineering, scaffolds with a porosity of over 70% are acceptable and ideal for the growth and nutrition of the cell 23. One of the important parameters in porous scaffolds is the correct and timely degradability. At the same time, as the texture is formed, the scaffold should be removed and replaced with natural texture. In fact, a biomaterial tissue must first be connected to the cell and then slowly destroyed, so that the mechanical properties of the implant are maintained and can support the regeneration process 24–26. In the slow degradation of tissues, an increase in tension leads to several damages to the architecture. Surface energy and the wettability of biological materials affect their biocompatibility. In this study, the morphology of the scaffold with a high porosity range and a suitable mechanical reaction was observed. The great migration, growth, and adhesion of the host tissue to the scaffold lead to great biocompatibility of scaffolds. Adhesion and absorption of proteins on the scaffold are also essential for cell adhesion. In this study, it was observed that the moderate levels of wetting (40–60*°C*) have the highest levels of protein absorption on the surface and thus have the highest levels of cell adhesion. Hydrophilic surfaces have higher protein absorption than hydrophilic surfaces, and thus, cells tend to be more adhesive to relatively hydrophilic surfaces.

**Table 1.** Obtained porosity (%) results from Image J and Archimedes’ principle for fabricated porous bio-nanocomposite scaffold using freeze drying technique with various amounts of Zr

**Sample**	**Porosity (%)**
**0% Zr**	77%
**10%Zr**	79%
**20% Zr**	83%
**30% Zr**	88%

### X-ray diffraction analysis

**Figure 3.**
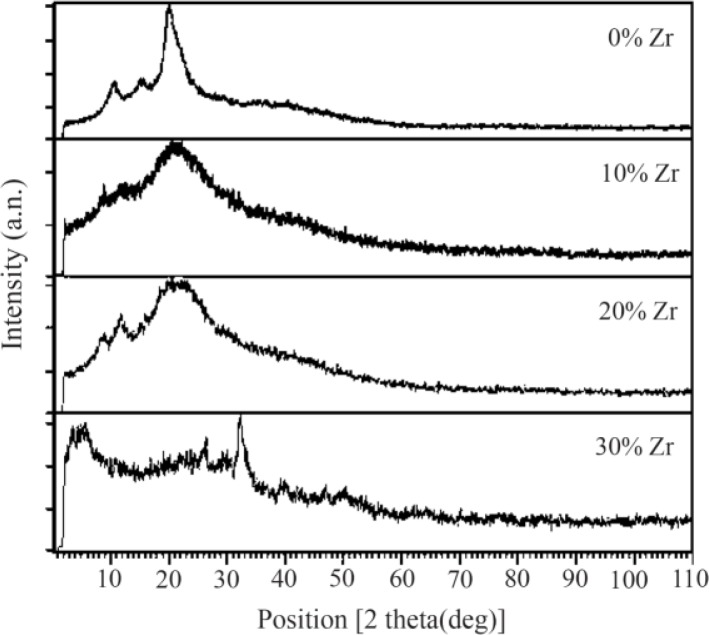
XRD pattern of samples with 0, 10, 20 and 30 wt% of Zr in the CS-HA composite powder.

The XRD patterns of the samples with various amounts of zirconium are shown in figure 3. The results show that if the scaffold stays in the in-situ environment, due to the formation of sediments on the scaffold and the mineralization of its surface over the time, it will be more robust. According to analysis, pure CS does not show any dispersion courier in the range of 40 to 90 degrees of 2θ. However, when zirconium is added to the matrix, the peaks shift and grow. The XRD results show a series of peaks at about 22 degrees, 25 degrees, 28.2 degrees, 31 degrees, 35 degrees, and 53 degrees. These peaks confirm the presence of a crystalline HA and zirconium in the network throughout nanocomposites. These peaks are related to the crystalline region of zirconium nanoparticles. The intensity of these peaks increased from sample 1 to sample 4 due to the addition of zirconium to the matrix polymer in the porous bio-nanocomposite structure. In the XRD pattern of CS curve, two crystalline peaks at 2θ were 10 and 30 degrees, which is consistent with the standard gelatin model. In the HA curve peaks in the 2θ, 23, 26, 29/3, 32, 46/6 and 49/00 can be seen that are in agreement with the standard peaks. These variations indicate a change in the nanocrystalline structure of the material, because Zr is a completely amorphous material; only a weak broad peak of about 2θ is 22 degrees in the XRD test.

### Mechanical properties

**Figure 4.**
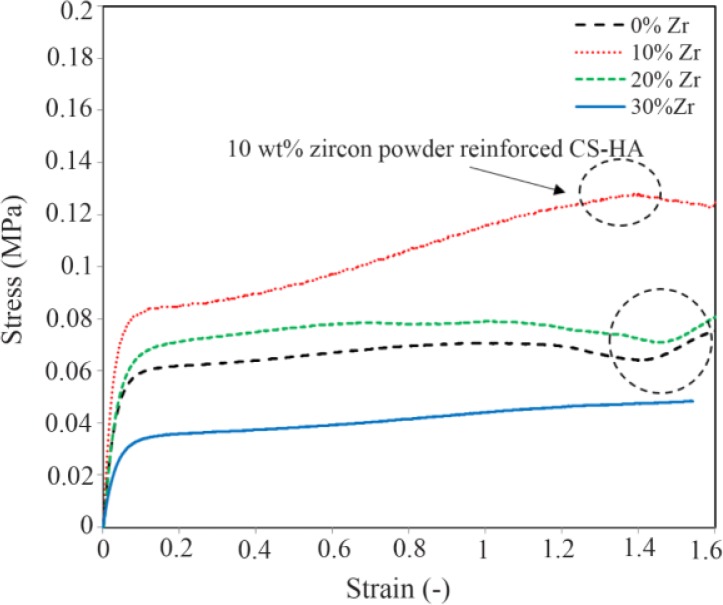
The stress-strain diagram of the samples with 0, 10, 20, and 30 wt% of Zr powder in the porous CS-HA scaffold.

**Figure 5.**
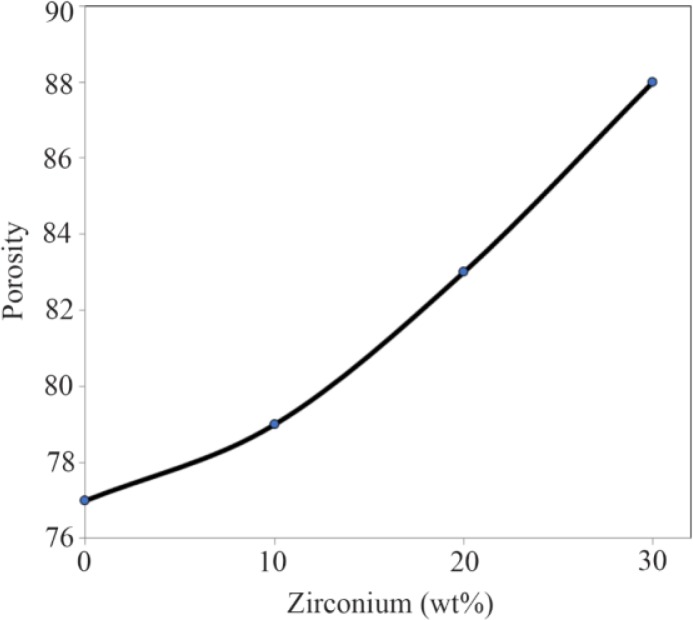
The porosity changes of the samples with 0, 10, 20, and 30 wt% of Zr in the CS-HA composite powder.

Evaluation of the tensile analysis, the elastic modulus and tensile strength of each sample is indicated in figure 4 from the slop of the stress-strain diagram. As it is illustrated, the highest elastic modulus and tensile strength was obtained for the specimen with 10 wt% of Zr. According to the diagram, with the increase in the amount of Zr nanoparticles, the modulus of elasticity and tensile strength of the samples improved. Elastic modulus increased from 1.6 to 3.1 *(MPa)* which is within the range of the elastic modulus of natural spongy bone. However, if the scaffold stays in the in-situ environment, the scaffold will be more robust due to the formation of sediments on the scaffold and the mineralization of its surface over the time. In all tensile stress curves, the stress-strain diagram can be divided into three parts 27–29. First, there is elastic region where strain is directly proportional to stress and increases with strain as the strain increases linearly. Second, there is elastic-plastic region where the change of strain does not have a linear fit with stress and third is the compaction or condensation region that occurs after initial loss in stress 30. In general, the mechanical properties of scaffolds can depend on crystallization. As expected, with the increase of zirconium to 10 wt%, improvement in the mechanical properties of the composite is observed and by increasing the weight ratio, the trend of increasing is reversed, such that in the specimen with 30 wt% of zirconium, the elastic modulus and tensile strength is less than the neat specimens. This mechanical behavior can be attributed to the porosity of the scaffold, which is another factor affecting mechanical properties of the scaffolds (Figure 5). This means that high porosity reduces mechanical properties in the scaffold.

### Bioactivity and biodegradability of composites

**Figure 6.**
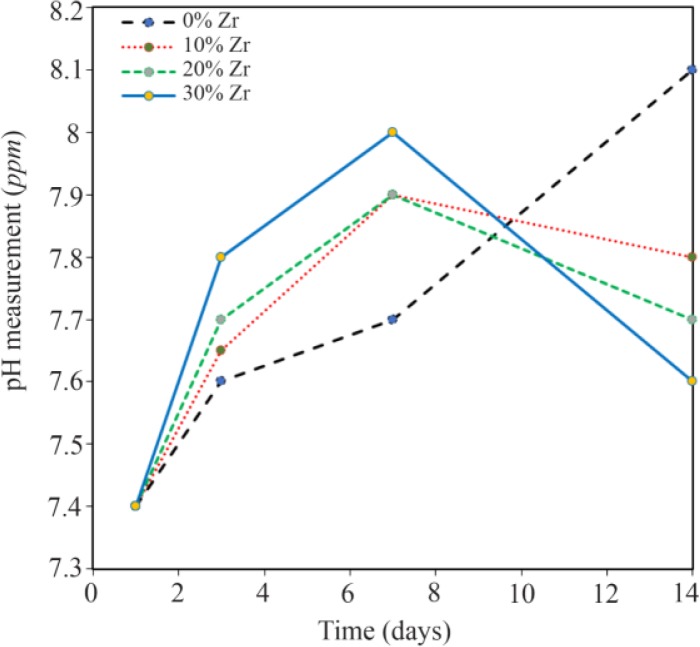
The pH changes of the samples with 0, 10, 20, and 30 wt% of Zr in the CS-HA composite powder.

Figure 6 shows the changes of pH in the samples after soaking in the SBF for 14 days in the water bath condition. Suitable humidity for nanocomposite scaffolds that are used for tissue engineering can provide nutrition and cell growth. In addition, synthesized scaffolds need to have structural stability in the human body. Therefore, the swelling behavior of the samples was studied in SBF to evaluate the regeneration of the scaffold within changes in pH value as shown in figure 5. As can be seen, the sample with 10 wt% of Zr had the highest stability and the lowest pH changes. As a result, it can be a suitable candidate amongst the rest of the samples for medical use.

**Figure 7.**
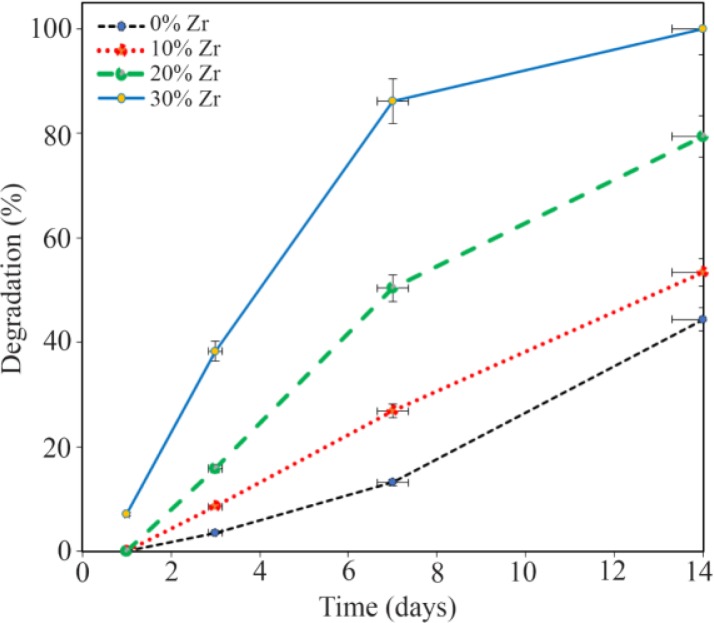
The degradation of the samples with 0, 10, 20, and 30 wt% of Zr in the CS-HA composite powder.

As shown in figure 7, the percentage of weight loss increases with increasing zirconium content in the scaffold, so that after 14 days, the sample with 30 wt% Zr is completely degraded, which is not a favorable result for this sample. It can be related to the presence of hydrophilic areas in the scaffold, which is justified due to the high-water capacity of these scaffolds. According to microscopic images, the porosity of the scaffolds containing zirconium is greater than the pure scaffold, so they have more water content and thus more weight loss. Therefore, it can be concluded that scaffolds with a higher percentage of zirconium have more weight loss.

### Cytotoxicity of prepared scaffolds

**Figure 8.**
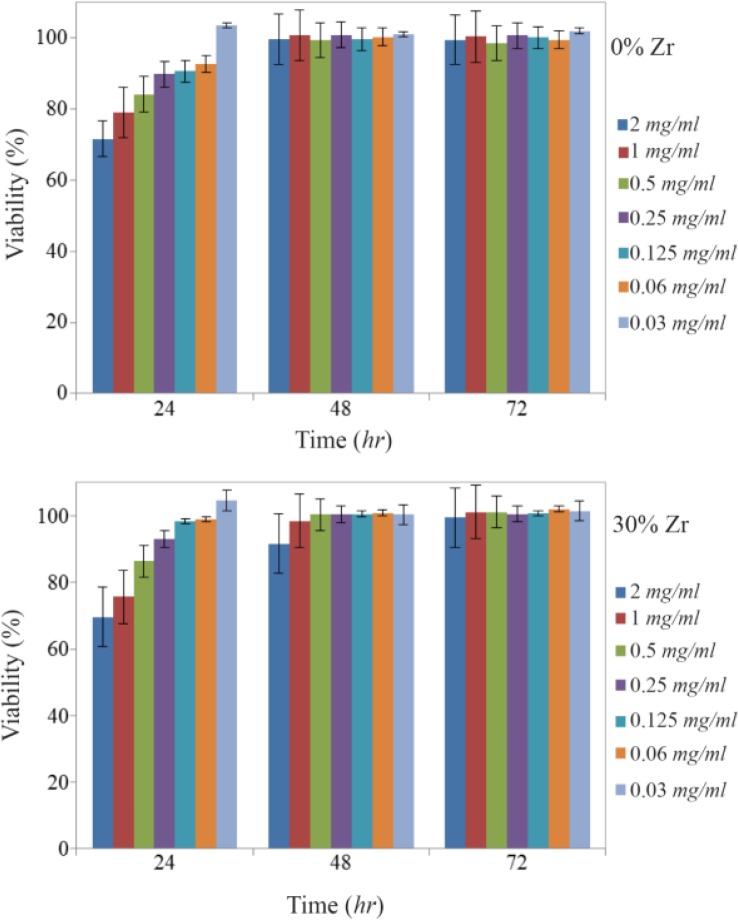
MTT assay of HuGu cells after 24, 48 and 72 *hr* with different concentration of scaffolds with a) 0 and b) 30 wt% of Zr.

**Figure 9.**
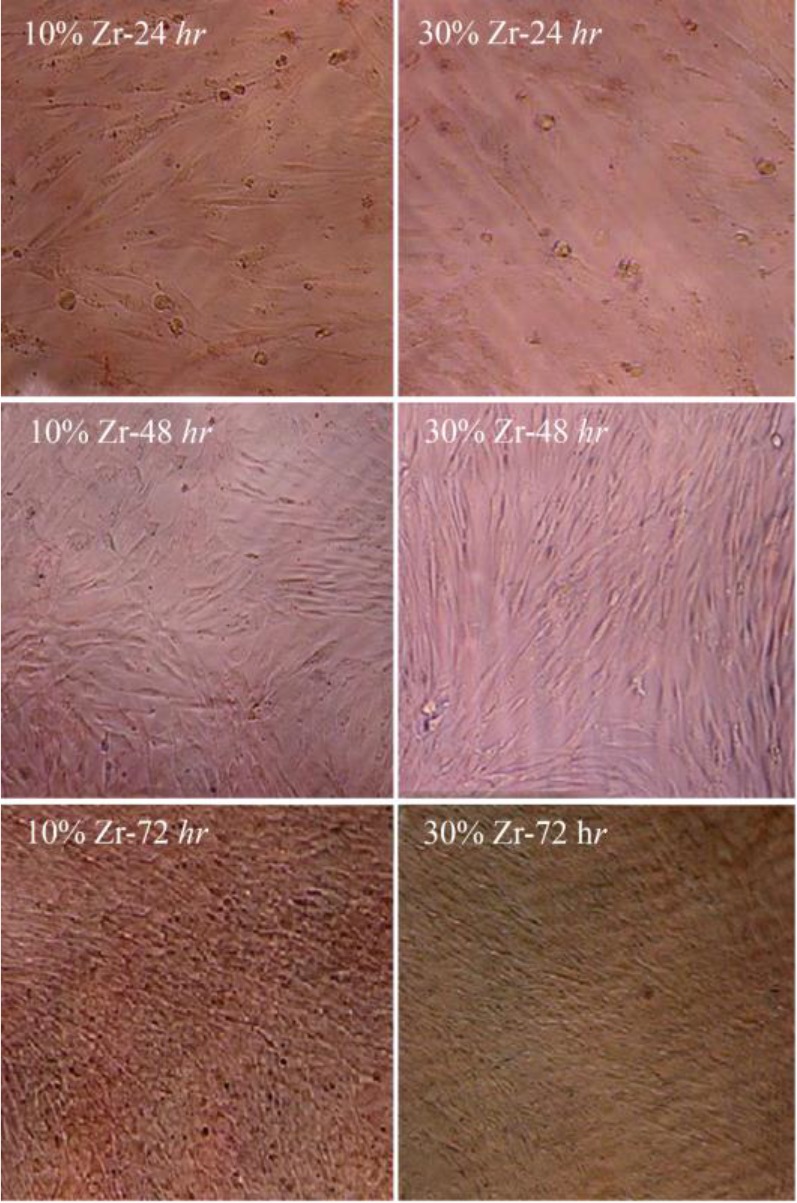
Optical images of the cell morphology in the presence of 10 wt% and 30 wt.% of zirconium in the CS-HA composite scaffolds extracts with the concentration of 0.5 *mg/ml* of PBS.

Briefly, the cell culture test for bone replacement has been reported in the cell environment using MTT assay. As shown in figure 8 (A, B), in the first 24 *hr*, even in higher concentrations of extracts, the biocompatibility was less than 100%, but after 48 *hr*, it had a remarkable improvement, and in nearly all concentrations, it was almost 100% and remained well maintained 31–33. The results indicate that the tested scaffold is not only highly biocompatible, but also a biocompatibility bias of between 24 *hr* and 48 *hr* shows that it has a positive effect on cell growth. In the first 24 *hr*, the cell viabilities of samples containing Zr were somewhat better than the cell viability of the neat sample due to the presence of more zirconium that provides better conditions for biocompatibility, which shows its compatibility with the body’s internal tissue. In addition, the optical images of the cell morphology of composite scaffolds extract with a concentration of 0.5 *mg/ml* of PBS in figure 9 shows that densely arranged cells were increased and they seemed to be piled up on each other through the time which approves that the results of the MTT assay in figure 8 are precise. The results indicate that extracts of the prepared 3D nanocomposite scaffolds showed sufficient cell viability for all concentrations. The good cell viability in an MTT assay indicates a high proliferation rate 9. As a result, nanocomposite scaffolds are ready-made, three-dimensional, and suitable for bone replacement with the ability to distribute drug-treated surfaces.

## Discussion

**Figure 10.**
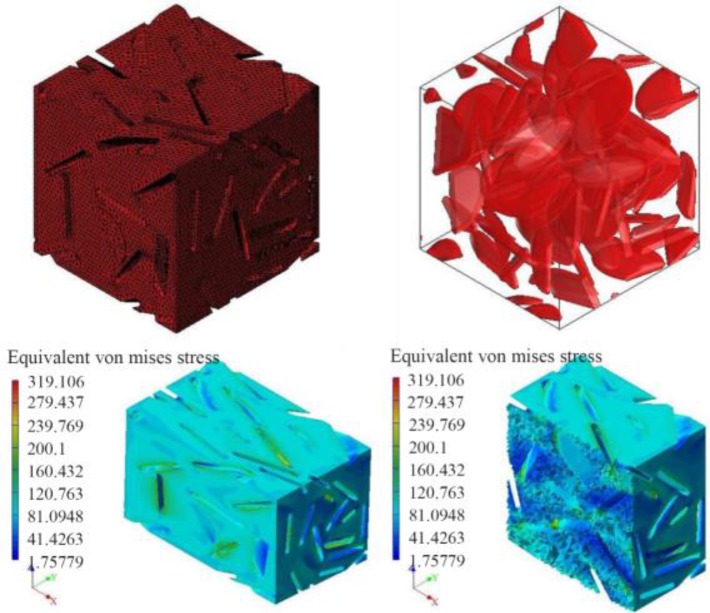
The macrostructure porosity of the designed porous bionanocomposite containing cell morphology in the presence of zirconium powder in the CS-HA.

**Figure 11.**
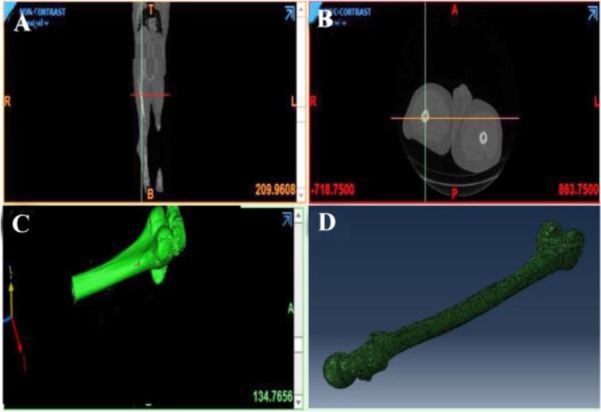
The femur design using CT-scan and MIMICs software to evaluate the porosity of the bio-nanocomposite.

As it is seen in figures 10 and 11, the fabricated porous bio-nanocomposite using freeze-drying technique was simulated in the ABAQUS software using MIMICs and CT-scans to evaluate the porosity and geometry of the tissue before applying in human body 34–36. The analysis indicated that the architecture of synthetic nanocomposite is similar to the human’s bone femur and can be a potential choice for bone implantation. Also, the analysis proved that the porosity and compression strength of the bone femur is nearly like a spongy bone regarding the medium pressure that can be tolerated during the analysis in the software. The mechanical performance of the articular cartilage is affected by a variety of factors such as cartilage and bone cortical density 37–39. The heterogeneous distribution of proteoglycans and collagen fibers in the direction of articular cartilage thickness leads to tissue formation with variable properties in the depth direction 40,41. These properties directly affect the amount of stress and variation in the cartilage. The observation of ABAQUS results shows that different properties and complex geometry of the components were neglected; therefore, the purpose of this study was to create a close-to-anatomy model of articular cartilage to simulate its behavior under dynamic load in the step-wise phase of motion which was successfully performed.

## Conclusion

In this research, a biodegradable nanocomposite containing CS, HA, WS and zirconium nanoparticle was made for the first-time using a freeze-drying technology. The SEM images showed interconnected and porous scaffolds with the presence of the zirconium nanoparticles. Using these images, it was determined that increasing the zirconium increases the porosity. The use of freeze-drying method caused porosity to be regular and the porosity size was in the appropriate range for the growth of bone cells. Afterward, the mechanical properties of the samples were examined and the obtained results indicated that as zirconium increased to 10 wt%, improvement in the mechanical properties of the composite was observed and the increasing trend continued as the weight ratio increased to 20 wt%. Also, the sample with 10 wt% zirconium has suitable bioactive behavior in the simulated body fluid. Except for the sample with 30 wt% zirconium, the rest of specimens possessed an acceptable biodegradability in the PBS. Subsequently, an MTT assay showed that prepared nanocomposite scaffolds were nontoxic due to the basic nature of the starting materials. Based on the obtained results, the specimen containing 10 wt% of zirconium is the most suitable candidate for bone tissue engineering application among the rest of specimens based on mechanical and biological tests.
